# ‘Once you've opened that can of worms’: qualitative study to understand why liaison psychiatry staff are not asking about domestic abuse following self-harm

**DOI:** 10.1192/bjo.2024.779

**Published:** 2024-10-11

**Authors:** Duleeka Knipe, Alison Gregory, Sarah Dangar, Tim Woodhouse, Prianka Padmanathan, Nav Kapur, Paul Moran, Jane Derges

**Affiliations:** Population Health Sciences, Bristol Medical School, University of Bristol, Bristol, UK; South Asian Clinical Toxicology Research Collaboration, Faculty of Medicine, University of Peradeniya, Sri Lanka; Alison Gregory Consultancy, Bristol, UK; School of Policy and Global Affairs, City St George's, University of London, London, UK; Suicide Prevention Programme, Public Health Department, Kent County Council, Maidstone, UK: and Centre for Health Services Studies, University of Kent, Canterbury, UK; National Confidential Inquiry into Suicide and Safety in Mental Health (NCISH), Centre for Mental Health and Safety, School of Health Sciences, University of Manchester, UK; Mersey Care NHS Foundation Trust, Manchester, UK; NIHR Greater Manchester Patient Safety Research Collaboration, University of Manchester, Manchester, UK

**Keywords:** Self-harm, qualitative research, suicide, mental health services, psychiatric nursing

## Abstract

**Background:**

Domestic abuse is a significant risk factor for self-harm and suicide. A large proportion of people presenting to healthcare services following self-harm have experienced domestic abuse. In the UK, routine enquiry for domestic abuse is recommended for people who present having self-harmed, but evidence indicates that this is not happening.

**Aims:**

An exploratory qualitative study to explore liaison psychiatry staff experiences of asking about domestic abuse, including the barriers and challenges to asking.

**Method:**

Semi-structured qualitative interviews with active adult liaison psychiatry staff in the UK. Recruitment was via online platforms and professional networks. A reflexive thematic analysis of the narratives was carried out.

**Results:**

Fifteen participants were interviewed across a variety of disciplines (ten nurses, four doctors, one social worker). The generated themes include the following: asking about domestic abuse – the tension between knowing and doing; ‘delving deeper’ and the fear of making things worse; the entanglement of shame, blame and despondency; domestic abuse was different from other clinical problems (mental illness/substance misuse); and biases, myths and misassumptions guiding practice. Participants indicated the need for better training and education, and clear protocols for eliciting and acting on disclosures.

**Conclusion:**

There is a clear need to improve the support offered to victim-survivors of domestic abuse who self-harm and present to healthcare services. National implementation of education and training to better equip liaison psychiatry teams with the skills and knowledge to sensitively support victim-survivors of domestic abuse is required.

Globally, more than 700 000 people die by suicide every year, with a further 14 million people engaging in acts of self-harm.^1,2^ Previous self-harm is a well-established risk factor for future suicide. Around one in two people who self-harm and present to services, and one in three people who die by suicide, have been affected by domestic abuse.^3–5^ Furthermore, people who experience domestic abuse and are in contact with mental health services before subsequently dying by suicide are more likely to have previously self-harmed than those who have not experienced domestic abuse either as a perpetrator or a victim.^6^

Victim-survivors of domestic abuse identify healthcare professionals, particularly doctors, as people from which they would most likely seek support from.^7^ Routine enquiry, alongside a well-trained clinical team, can increase referrals to specialist services to support those experiencing domestic abuse.^8^

## Existing guidance

The National Institute for Health and Care Excellence (NICE) guideline for the assessment, management and prevention of self-harm in England recommends that ‘[a]ll staff who have contact with people who self-harm should ask about safeguarding concerns, for example, domestic abuse … ’.^9^ Responsibility for health is devolved to individual UK nations who make their own decisions about guideline implementation, but key elements of NICE guidance are typically adopted. In Scotland the Scottish Intercollegiate Guideline Network (SIGN) produce their own guidelines, but there is no separate SIGN guideline for self-harm. In addition, in the NICE domestic violence and abuse quality standards, one of the basic quality indicators is that ‘[p]eople presenting to frontline staff with indicators of possible domestic violence or abuse are asked about their experiences in a private discussion’.^10^ Suicidality and self-harm are also stipulated by NICE as key indicators of domestic abuse victimisation.

## What this study adds

Despite the strong link between self-harm and domestic abuse victimisation and perpetration, and explicit NICE recommendations, data from a self-harm surveillance register in England found that only one in five people who presented to services following a self-harm incident and received a psychosocial assessment had any record of a discussion about domestic abuse.^5^ This proportion is likely to be even lower in the approximately 40% of people who do not receive a psychosocial assessment after self-harm.^11^ Practitioners working in liaison psychiatry routinely encounter people with complex health and social needs. Yet, it is unclear why mental health professionals, who are specifically trained to discuss difficult topics, are not routinely enquiring and documenting domestic abuse following a self-harm presentation to services. We aimed to explore liaison psychiatry staff experiences of asking about domestic abuse, including the barriers and challenges to asking.

## Method

### Design

Semi-structured qualitative interviews were conducted to gather the perspectives and experiences of clinicians currently working in UK liaison psychiatry teams.

### Recruitment

Recruitment took place between September and October 2022. Participants were asked to volunteer to take part via social media (Twitter) and professional networks, in addition to a request posted to members of a LISTSERV for UK liaison psychiatry staff. UK healthcare professionals were included if they currently worked in adult liaison psychiatry services, and regularly supported people who presented following self-harm. Twenty-two people volunteered to take part, and we adopted an information power approach to inform our decision about when to stop recruitment,^12^ that is, a parallel process that assessed the characteristics of the recruited sample, and the depth and quality of the data gathered in relation to the specific study question, to inform when to halt recruitment. Given the specificity of the study aim, the variation of experiences represented in the sample and the quality and depth of dialogue, recruitment was halted at 15 participants. All interviews were recorded and transcribed using Microsoft Teams software.

### Consent

All recruited participants were sent information about the study in advance and consent forms were completed electronically before the scheduled interview. Interviews were conducted online using the Microsoft Teams platform, and participants were given opportunity at the start to ask any questions and verbally reconfirm consent.

### Ethical approval

The authors assert that all procedures contributing to this work comply with the ethical standards of the relevant national and institutional committees on human experimentation and with the Helsinki Declaration of 1975, as revised in 2013. All procedures involving human participants/patients were approved by the University of Bristol's Faculty of Health Sciences Research Ethics Committee (FREC ref. 12263).

### Interviews

J.D. conducted the interviews using a semi-structured topic guide developed through discussion between D.K. and J.D. (Supplementary Material available at https://doi.org/10.1192/bjo.2024.779). The topic guide was structured around questions relating to how, when and who should ask about domestic abuse when someone presents following self-harm, as well as exploring existing knowledge, training and experiences of domestic abuse. From here on in when we write about domestic abuse enquiry, we are referring to both domestic abuse victimisation and perpetration unless specified. The topic guide was used to steer the interview but was not adhered to rigidly to allow for flexible exploration of emerging topics during the interviews.^13^ All participants were offered a £20 ‘thank you’ shopping voucher. Interviews ranged in length from 30 to 60 min.

### Researcher reflexivity

J.D. is a social scientist and led the interviews and analysis. She identifies as a White woman and previously had a career as a mental health occupational therapist. D.K., who supervised the study and contributed to the analysis, identifies as a South Asian woman and has family members who are victim-survivors of domestic abuse, and who have self-harmed because of this. Both J.D. and D.K. have worked in the field of suicide and self-harm research for over 10 years.

### Analysis

Transcripts for the interviews were automatically generated by Microsoft Teams software and were checked and corrected by J.D. Reflexive thematic analysis was used to analyse the data. This approach enables the exploration of data and the generation of key themes to help us understand professionals’ views and experiences related to the topic.^14^ Analysis was conducted concurrently with the interviews so that areas for further exploration could be investigated in the interviews. J.D. and D.K. initially read all the transcripts several times to become familiar with the content, before randomly selecting three transcripts to code independently. In this inductive process, relevant sections of text were identified via a process of line-by-line reading, coding and organisation of codes into a series of broad categories. J.D. and D.K. refined and synthesised the codes and categories through discussion. J.D. used these refined codes to complete the coding of the remaining transcripts. From the coded transcripts, themes were developed in relation to the research topic of interest. We present quotes under pseudonyms.

## Results

Fifteen professionals working within UK National Health Service (NHS) liaison psychiatry services were interviewed: nine mental health nurses (MHNs), two consultant psychiatrists, two junior psychiatrists, one nurse working with older adults and in community services and one social worker (Table 1). Length of practice varied between 3 months and 30 years. Participants were geographically dispersed across England and Scotland. The perspectives from which participants chose to respond to interview questions varied according to professional experience and role. For example, consultant psychiatrists and senior nurses tended to discuss the topic in general from their perspective as team leader/manager, but also spoke about individual experiences and were able to give case examples, whereas junior psychiatrists and nurses spoke about their own clinical experiences of asking patients about domestic abuse and case examples. In addition, four professionals talked about their own personal experiences of domestic abuse victimisation and, for two participants, these experiences had led to their interest in the topic. A few participants, particularly those with personal experiences of domestic abuse, found the topic difficult to talk about but were nonetheless keen to participate and were well supported throughout. Despite our enquiry being about people who experienced domestic abuse (which would include both victims and perpetrators), participants commonly answered questions with only victims in mind.

**Table 1:** Basic characteristics of study participants

Name^a^	Gender	Role	Years of experience
Karen	Female	Mental health nurse (community)	5–15
Debbie	Female	Social worker	<5
Jim	Male	Mental health nurse	15+
Paul	Male	Mental health nurse	15+
Maya	Female	Consultant psychiatrist	15+
David	Male	Consultant psychiatrist	15+
Ann	Female	Mental health nurse	15+
Wei	Male	Psychiatrist	<5
Aisha	Female	Mental health nurse	15+
Desiree	Female	Mental health nurse	<5
Rina	Female	Mental health nurse	15+
Tracey	Female	Mental health nurse	<5
Daisy	Female	Mental health nurse	15+
Kelly	Female	Mental health nurse	<5
Carla	Female	Psychiatrist	<5

a. Pseudonyms are used to protect the anonymity of participants.

It was apparent from people's narratives that participants responded in a variety of ways/took a variety of actions or decisions when they had concerns that someone was experiencing domestic abuse. From the analysis the following key themes were developed: (i) asking about domestic abuse: the tension between knowing and doing; (ii) ‘delving deeper’ and the fear of making things worse; (iii) the entanglement of shame, blame and despondency; (iv) domestic abuse was different; and (v) biases, myths and misassumptions guiding practice.

### Asking about domestic abuse: the tension between knowing and doing

All participants agreed that asking about domestic abuse was important and that this sat firmly within their professional role and remit, they did not routinely ask everyone, indicating that this routine enquiry was difficult in the time allowed for assessment and that participants did not know exactly what to do following a disclosure. Most participants reported selective enquiry about domestic abuse only in the presence of clear warning indicators such as signs of physical violence, secretiveness or an obvious expression of fear when talking about a partner or relative, or when domestic abuse was already documented in patient notes:

‘I think it's everyone's role, not just mine … . So, you know, when they're in triage and as they go through that pathway before they see us. But yeah, I mean, it's certainly something. It's not something I routinely ask. I have to be honest.’ (Jim, MHN)

Proformas and checklists for assessing suicide risk were generally unpopular and were not considered an effective tool in trying to understand suicide, self-harm, mental health or domestic abuse. The use of checklists tended to be more common among junior members of staff who potentially lacked experience and confidence:

‘Yeah, we use a checklist and, well, obviously we try not to be that prescriptive, but, yeah, it's because I'm relatively new as well, I think just for my own peace of mind, I like to use that and as a very like systematic solution focused person, I think that works well for me. But yeah, generally that isn't something that immediately comes up within one of those checklists.’ (Debbie, social worker)

If children were involved, questions about domestic abuse became more explicit and routine because of safeguarding concerns. A lack of confidence in knowing what to do once a disclosure was made was indicated as a barrier, despite all participants being able to cite resources available to them. Some participants suggested that a system which provided an easy set of instructions regarding what to do once a disclosure had been made would help to overcome this barrier, this was described as a ‘big red button on your computer’:

‘[B]asically having, you know, an entry portal …  to make it easy for clinicians to access the right information and to report in the right way.’ (Maya, consultant psychiatrist)

Participants indicated that hindrances to asking about domestic abuse also included not having sufficient time and privacy, especially in emergency departments where only a curtain separates bay areas, and there is typically no private room in which to talk. In addition, personal experience of domestic abuse victimisation affected likelihood of asking about domestic abuse, with effects dependent on whether the abuse was past (enquiry likely) or current (less likely). The concept of ‘opening a can of worms’ was mentioned by several participants in relation to the idea that identifying and carefully managing a disclosure of domestic abuse could be extremely time-consuming. A disclosure could trigger a cascade of events including decisions about contacting children's services, contacting police and the repercussions of this, and finding the person alternative accommodation – these were all cited as barriers to enquiry. This was particularly the case for professionals working in busy emergency departments, where the emphasis was on rapid assessment. Moreover, addressing domestic abuse victimisation was very challenging when a patient was unable to yet identify their experiences as abuse, when the domestic abuse involved more than one abuser, or when safety options were unavailable.

### ‘Delving deeper’ and the fear of making things worse

Participants reported that they used different enquiry methods for identifying domestic abuse but expressed concern that their questioning might cause distress and discomfort. Three main methods for identifying experiences of domestic abuse were reported: waiting for the patient to disclose; direct questioning; and facilitating a disclosure. These first two methods were, however, used infrequently, with the facilitation of a disclosure being much more common:

‘I think generally things often come out during the assessment where there's something alluded to “Ohh, you know he wasn't a very nice person. I discovered it after I had our second child and I left him” and I might then sort of use that as a cue to sort of pursue that … . I'm thinking really hard here, because I thought that I was actually quite good at asking this question and I'm realising that often it's in response to something that the client has revealed that I would then go on to ask.’ (Aisha, MHN)

Many participants talked about taking a history, which includes questions about family and relationships, and looking at past documentation in patient notes, especially previous contact with services. Participants indicated that they usually raise the topic by asking questions such as: ‘Can you tell me about your relationships?’, or ‘How are things at home?’. People felt that this less direct approach was a helpful tactic to identify abuse without the patient feeling confronted, frightened, offended, uncomfortable or triggered. Participants described approaches that were reportedly used across the represented disciplines:

‘ … I wouldn't sort of go in at the deep end and say, are you experiencing domestic violence? I look out for sort of signs that may, I don't know, be giving it a kind of clue or a hint that it might be happening. So, say if they were saying that they have a lot of arguments with their partner or something like that, then I would start digging down a bit deeper.’ (Kelly, MHN)

The few participants who reported more direct questioning, used questions such as: ‘Has there ever been any incidents of violence?’ (Desiree, MHN). When time allowed, opportunities to build rapport were considered important in advance of asking more direct questions.

In participant narratives a heavy reliance on ‘looking for red flags’, ‘picking up’ signals about potential abuse were apparent. References were made to ‘cues’ and ‘hints’, or patients appearing secretive, or talking in a particular way about partners/relationships. Alongside looking for physical marks of bruising or injury, most participants described using intuition and, with experience, felt that this was a reliable and trusted skill when asking about domestic abuse. However, self-harm, one of the indicators highlighted in the NICE domestic violence and abuse quality standards, was not spoken about by participants in of itself as a ‘red flag’.

Participants described that once a disclosure had been made, it was possible to explore in more depth with patients, allowing a greater level of ‘delving’. This delving was seen as carrying some risk, for example, the patient might be further upset, or feel shame about their experiences, or traumatic memories of past abuse might be triggered. Participants also had concerns about the potential for patient suggestibility:

‘A victim of domestic violence, they're scared already. You know they are scared of whatever is happening, but they don't necessarily have insight into it. They just know what's happening is wrong and maybe they don't know that, so when you ask these questions, there is a risk of them, their mental health becoming worse, and especially in terms of like suicidality, low mood, well, also like feeling more unsafe … I try not to do it too soon because as I said, you know, it's a very sensitive topic and  … .I often find that you have to be careful in terms of putting ideas into people's heads.’ (Tracey, MHN)

In terms of how professionals probed, participants indicated that considerable skill and sensitivity were employed, in particular probing beyond the presenting issue to understanding the context of the person's life:

‘Certainly, in my own practice, the majority of women that I've seen, are there because they probably poisoned themselves. My own style is rather than just talk specifically about the moment of the overdose, I take a person back to kind of – OK, tell me tell me about that morning, start there. And get them to start narrating the story. It's hopefully helpful for them to narrate their story and someone listening fully to what's going on.’ (Paul, MHN).

### The entanglement of shame, blame and despondency

Several participants spoke of the potential for patients to feel shame, or guilt, associated with their experience of domestic abuse victimisation and their narratives indicated how this could be enmeshed or entangled with their own judgements, blame and sense of despondency about the situation. The notion of shame was raised in response to questions about barriers to asking about domestic abuse:

‘If they feel like they're uncomfortable to talk about, or shamed – actually, often people are quite embarrassed get the sense there's often a lot of guilt, or a sense of “it's my fault, I must have done something, I deserve it”. So, you get that sense I think with people when they're embarrassed or they're ashamed or they're afraid.’ (Ann, MHN)

Several participants gave an indication that feelings of shame may, in fact, be an assumption or projection on their part or connected with their own thoughts about the patient's decisions and choices:

‘I think it is uncomfortable to ask about. I think that sometimes we can project our own feelings of shame on to our patients, and it stops us asking sensitive questions.’ (Maya, consultant psychiatrist)

‘There is still maybe shame around it and that if we ask about it and find that it's there, then we'll be confronted, you know, the clinician and the patient with the fact that the patient is choosing to still not just up and leave and go to the police, and, no matter the kind of the reasons why, that feels so difficult and impossible, and all sorts of things. I guess there's still maybe that element of shame there, which I think carries across to the professionals somehow.’ (Wei, psychiatrist)

In addition, part of the complexity in asking about domestic abuse victimisation was the despondency participants described feeling when a patient who was experiencing abuse remained with or returned to a relationship with the abuser – a phenomenon that was commonly reported. Although participants were able to acknowledge that leaving an abusive relationship was neither easy nor safe, those who felt they had put in work to support and advise victim-survivors, often over long hours, could end up feeling despondent:

‘ … a feeling that [my colleagues] want to help but don't know how they do. They don't want to open the can of worms because then there's worms all over the floor and they have to pick them up.’ (David, consultant psychiatrist)

### Domestic abuse was different

Participants working across all settings highlighted a distinction between domestic abuse and other vulnerabilities (i.e. mental ill health and substance misuse), feeling that domestic abuse was simply different. For example, although the management of substance misuse was deemed to form an integral part of liaison psychiatry training, people felt that ‘there isn't a sort of ingrained culture about asking about domestic abuse’. Tackling domestic abuse, and understanding the complexity of a patient's situation, was also reported as being more complicated:

‘[Domestic abuse is] much more difficult than working out the clinical problem. Much more difficult, you know, if there is a sort of a domestic abuse situation going on in a patient you're looking after – that patient will take you, you know, order of magnitude, more time and energy than a patient without that as part of their picture… We'll take a huge amount more time than managing even a really complex clinical problem.’ (Maya, consultant psychiatrist)

‘I guess I know on a theoretical level that that is just as important as asking somebody about drugs and alcohol, which is something we routinely do …  I think is it almost when somebody is either the perpetrator or the victim of domestic violence, I imagine they probably, quite wrongly, feel a sense of agency within that. Whereas with drugs and alcohol, it's kind of, it's almost like “yes” or “no”, it's like do you use drugs, alcohol? “Yes, I do, no I don't”, whereas domestic violence is so much more …  complicated, I suppose, and insidious perhaps … and so it was a bit more of an intrusive question and people might struggle to answer that.’ (Wei, psychiatrist)

Related to this perceived distinction between domestic abuse and mental health issues was the acknowledgement that the current training and culture around asking about domestic abuse needed improvement. In addition, when presented with the statistics and associations between domestic abuse and self-harm, whilst most participants were not surprised, many said that the strength of the associations was greater than they had anticipated/expected and was not reflective of their own experience working in liaison psychiatry:

‘As for why it's not something that I instantly think of, I don't know whether it's just sort of like lack of that having been covered like medical school teaching since being a doctor and, I'm yeah, I'm not entirely sure. Just it never never springs to mind.’ (Carla, Psychiatrist)

‘That statistic which is, you know, as I said quite eye opening. So perhaps that is a question I will ask more now you know, regardless of whether there are concerns or whether there's really, you know clear evidence.’ (Jim, MHN)

Furthermore, related resources and their accessibility really differed across regions with variation in how well resourced participants felt in terms of colleagues who could advise, and routes for specialist referral. Existing resources were said to be effective, and independent domestic violence advisors (IDVAs) were frequently referenced as a well-used resource where expert knowledge could be gained when needed. Although, again, participants indicated a perceived difference between domestic abuse and other vulnerabilities, regarding the resources available:

‘So I think inevitably I feel more confident as a practitioner because I know I can just go upstairs to my office and say to my lovely alcohol colleagues: “by the way this person is misusing alcohol or substances” and they'll be able to kind of tell me exactly what to do and what the protocols are and whereas I suppose we don't have like a domestic violence liaison service. So, we do have like a hospital IDVA … . And she's really helpful and we can talk to her if we need to, but obviously it's not quite the same.’ (Debbie, social worker)

### Biases, myths and misassumptions guiding practice

An awareness of personal biases when asking patients from diverse backgrounds about domestic abuse were openly acknowledged by most participants. All participants mentioned a concerted desire not to treat or consider patients differently, and yet also talked about potential areas of bias, both in terms of whether and how domestic abuse was addressed. Even when able to freely acknowledge that domestic abuse crosses generations and genders, some participants reported more commonly considering and asking about domestic abuse victimisation with female patients:

‘I've never met a man who has been abused.’ (David, consultant psychiatrist)

‘Definitely I think I'd be more inclined to think that this – a woman is more likely to be experiencing that than a man and then when it comes to people that are … .and you know, transgender or gender fluid or gender non-binary, I think I'm definitely more concerned about it then as well.’ (Debbie, social worker)

‘I guess my professionally sceptical head goes – you know, any man that claims he's the victim, I'm automatically a bit suspect about.’ (Paul, MHN)

Other participants were, however, able to give examples of male patients they had seen, who had experienced domestic abuse victimisation, specifically in the context of same-gender relationships. With regards to ethnicity, participants voiced assumptions about domestic abuse victimisation being more prevalent or more ‘typical’ among particular ethnic minority groups, including ideas about domestic abuse being an aspect of a cultural ‘norm’ in more patriarchal communities. Whilst most participants felt that domestic abuse was not acceptable in any situation, they also indicated a level of discomfort and concern that they might ‘misinterpret’ a situation:

‘And I think the culture is quite difficult because with the different cultures and what is the norm for somebody in their own culture, I would probably raise an eyebrow, and think – whoa.’ (Rina, MHN)

‘I think in regards to ethnicity, again, trying to be aware of that – bit culture, bit ethnicity, bit religion, people don't understand that people don't necessarily relate to each other in the way that I might relate to each other, and what is their norm. That doesn't make it right if someone's not being very nice to you, but you know, in some cultures where males may be seen in a more dominant position or whatever that might be, on this I think in trying to understand that I might miss it, misinterpret a situation because I don't understand someone's ethnicity or not well enough … ’ (Kelly, MHN).

Additional factors related to a patient's age and socioeconomic status were raised. Older age patients were less likely to be asked about domestic abuse victimisation, unless the clinician worked in older age psychiatry, and this was compounded when a patient had dementia. Participants also reported that they were less likely to ask patients who they perceived as ‘middle class’ because of feelings of intimidation, and an assumption that ‘middle class’ professional patients were less likely to experience domestic abuse victimisation.

## Discussion

The findings from our study indicate that whilst the importance of asking about domestic abuse in the context of self-harm is acknowledged by liaison psychiatry staff in the UK, routine enquiry does not happen in practice, and there are a number of barriers to more general enquiry. This is, in part, because domestic abuse is seen as *different* from other vulnerabilities, for example substance misuse and mental illness, and there is no embedded culture to explore domestic abuse as part of routine care.

Personal biases, myths and misassumptions act as strong barriers to asking about domestic abuse, as do clinicians’ (mis)perceptions of victim-survivor shame, and guilt. Fear of ‘causing offense’, ‘frightening’ someone or ‘triggering’ also limits clinicians’ willingness to ask about domestic abuse, in addition to concerns that supporting someone experiencing domestic abuse will take up significantly more resources than other complex clinical problems. These apprehensions about workload are compounded by the despondency clinicians feel when they have invested a lot of time in supporting a victim-survivor who subsequently returns to the abuser. Participant suggestions to improve clinicians’ enquiry about domestic abuse following self-harm include better training about the link between domestic abuse and self-harm, as well as opportunities to increase confidence around ways to ask, and how to deal with disclosures. The latter could be supported by prompts through computerised systems that provide a standard cascade of next steps and improved relationships with hospital IDVAs (where they are in place) to access support and advice. IDVAs are commissioned to provide specialist domestic abuse support within hospitals, yet based on our participant responses, it appears that the role of IDVAs may not be fully understood by staff and highlights another focus for future training initiatives. Consistent with evidence, most participants did not use standard checklists or proformas to assess risk given that (i) such checklists are known to be ineffective and (ii) they these may impede the building of rapport and therapeutic alliance, particularly with vulnerable individuals who are victim-survivors of domestic abuse.^15^ The use of a similar checklist approach to enquire about domestic abuse therefore is unlikely to be effective or appropriate. However, the absence of a prompting system appears to limit clinician enquiry about domestic abuse and may lead to this risk factor being overlooked during as part of patient risk assessments.

No participants raised the impact of disclosure of domestic abuse on the risk assessment. Victim-survivors of domestic abuse are unlikely to disclose violence and abuse unless directly asked,^16^ and are more likely to disclose to healthcare professionals. Victim-survivors have reported that barriers to disclosure to healthcare professionals include concerns over being judged, their experiences being minimised and perceived low capability of professionals to help them.^16^ In addition, it is important to note that leaving an abusive relationship is not the only positive outcome of a disclosure. Victim-survivors valued shared decision-making and feeling validated, without pressure to leave or press charges.^7^ A better understanding of this amongst liaison psychiatry staff might help ameliorate the feelings of despondency that were reported. Past research has highlighted that whilst direct questioning is preferrable, this would only be acceptable to victim-survivors if healthcare professionals acknowledged and addressed the patient's feelings of shame, as well as their autonomy and physical safety.^15^ The reluctance to enquire about domestic abuse in liaison psychiatry services is consistent with other specialisms (e.g. general practice, emergency department staff), with many of the barriers highlighted also being similar (e.g. fear of offending, lack of time, discomfort and frustration).^17^ Mental health professionals have also reported being reluctant to ask patients about domestic abuse because of fear of causing offence and resource limitations, as well as a lack of confidence – because of lack of training – in dealing with the aftermath of disclosures.^18,19^ Despite liaison psychiatry staff reporting barriers in asking related to concerns over the patient, victim-survivors want to be asked.^20^ Lack of training and education is a recurring barrier reported by healthcare professionals, in addition to an absence of standard protocols for dealing with disclosures.^17^ The training, educational and protocol barriers contribute to other barriers identified by health professionals, in particular clinicians’ lack of confidence. Greater integration of current practice guidance for mental health professionals to respond to domestic abuse is needed.^21^ This guidance includes how to ask and respond to perpetrators of domestic abuse, who have a considerably elevated risk of suicide,^22^ and require a different approach.

The biases and misconceptions around domestic abuse highlight the need for a greater generalised awareness of the complexity of domestic abuse, in particular for a specialism that frequently encounters victim-survivors whose extreme distress has contributed to them harming themselves. There appeared to be an assumption that abuse was mostly a woman's experience, which although generally true, in the context of self-harm, it is highly likely that this perspective meant some men were overlooked.^23^ Consistent with other mental health professionals, liaison psychiatry staff reported that ‘cultural norms’ amongst certain ethnic groups meant that enquiry was less likely,^18^ despite evidence indicating that ethnic minority groups are more likely to experience domestic abuse^24^ and thus a failure to enquire is likely to widen health inequalities. In addition, the assumption that people from a higher socioeconomic position are less likely to experience domestic abuse, which again is accurate,^23,24^ is unhelpful given that experiences of domestic abuse are still prevalent in these groups. Training to increase awareness is needed at the earliest possible stages in people's healthcare careers, ideally within university degrees with refresher training as part of continued professional development sessions. The current UK training received by medical students on domestic abuse has been found to be minimal and inadequate, and needs further development as a priority.^25^ Previous work has shown how educational programmes for health professionals have been effective in reducing the barriers identified in this and other related studies.^8^

As an important additional finding, four of the 15 participants in this study disclosed personal experience of domestic abuse victimisation. Although this study is small and exploratory, these figures are consistent with recent findings.^26^ Dheensa et al's^26^ systematic review highlighted the impact of personal experiences on healthcare professionals’ ability to enquire about abuse and to support patients who are victim-survivors of domestic abuse. The findings of our study support this. Any training, therefore, needs to be delivered sensitively. It is possible that by increasing domestic abuse training and education, the environment where staff work could become more supportive of disclosures by patients and staff.

Whilst domestic abuse increases the risk of self-harm in a similar way to substance misuse and mental ill health, the support and protocols following disclosure are not available or standard across different liaison psychiatry services. Participants indicated that it would be helpful having a metaphorical ‘red button’ to press, giving the clinician a cascade of actions to take when a disclosure is made. In addition, they would appreciate someone who was a specialist in domestic abuse within their liaison psychiatry team. These suggestions, alongside the above-mentioned need for training/education, are specific components of a previously trialled specialist domestic abuse training, support and referral programme for primary care that has been shown to be effective in improving referral and identification of domestic abuse.^8^ This would be a promising intervention for adaption and trialling with liaison psychiatry services across the country.

### Strengths and limitations

To the best of our knowledge this is the first UK-based study exploring the perspectives liaison psychiatry staff who support individuals who present to services following self-harm. Capturing the views and experiences of professionals is an important first step towards encouraging increased enquiry and support for victim-survivors. Since this was an exploratory study, the primary limitation is that the number of participants was small; we can thus only claim to have captured a subset of views and experiences. However, we were able to delve down and capture people's views in depth. The second, related limitation, is the relative homogeneity of participants regarding ethnicity (93% identified as White), despite efforts to recruit a more diverse sample. Interviewees self-selected and this might indicate invested interest in the topic. In common with other qualitative studies, a degree of caution should be exercised in considering the transferability of our findings beyond the context in which the data were collected.

In conclusion, there is a clear need to improve the support offered to victim-survivors of domestic abuse who self-harm and present to healthcare services. Given the prevalence of domestic abuse experiences in patients who self-harm, and the fact that without direct questioning disclosures are unlikely, there is a need for routine enquiry for all patients presenting with self-harm to be embedded into liaison psychiatry clinical culture. Clear protocols and care pathways are needed that are both well disseminated and integrated. To be effective staff need to trained and educated on how to best support victim-survivors. Training needs to not only equip staff in routinely asking about domestic abuse, but also how best to intervene effectively following a disclosure. This will include ensuring a greater awareness of the role of IDVAs and how to utilise them. The adaptation of existing interventions for clinical staff in other specialisms should be considered for staff working in liaison psychiatry, which need to be delivered sensitively to acknowledge that clinicians attending the training may also have been affected by domestic abuse (as victims or perpetrators) and may need to be signposted to, or provided, specific support.

## Supporting information

Knipe et al. supplementary material Knipe et al. supplementary material

## Data Availability

The data are not publicly available because of the potential to identify participants from interview responses, which would compromise the privacy of research participants.
