# Supplementary material for: ‘Once you've opened that can of worms’: qualitative study to understand why liaison psychiatry staff are not asking about domestic abuse following self-harm
Source: BJPsych Open. 2024 Oct 11;10(6):e177. doi: 10.1192/bjo.2024.779 (PMC11536313; doi:10.1192/bjo.2024.779)
Supplement: Knipe et al. supplementary material [file S2056472424007798sup001.docx]

**Liaison psychiatry staff: topic guide**

**Domestic violence and self-harm - why are we not asking about it?**

**Introduction**

Can you briefly tell me what your role is and how long you have been practicing?

1. **Talking about DV during assessments – who should ask, and when**

Recent data suggests 1 in 4 suicide deaths have been impacted by DV and roughly 1 in 2 people who attempt suicide have experienced DV? Does this match with your professional experience?

Is it your role to ask about DV when an individual presents following a self-harm/ suicide attempt (SH/SA)? If not, why not? Is there someone else who should be asking?

Is addressing possible DV a routine part of your assessment for individual’s presenting with SH/ SA? Or is it raised only when there is a suspicion of DV? Or if/ when the individual raises it?

Is talking about DV similar to addressing other mental health-related topics? If not, what might be different?

Is asking about DV the same conversation across genders? If not, what might be different?

Is talking about DV the same conversation across all ethnic groups? If not, what might be different?

1. **Initiating conversations about DV – how to ask**

What circumstances might prompt you ask an individual presenting with SH/SA about possible DV? (e.g., when there are strong indicators, such as physical marks or when the person is unable/ unwilling to tell you what is worrying them? Any other signs?)

Are there circumstances when you wouldn’t ask about DV, and what are your reasons?

Do you perceive any risks to asking about DV?

How might you ask/ address DV? Please give general examples...(i.e. asking directly/ talking more generally about the topic/ giving written handouts with support information)

How confident/ comfortable do you feel asking someone about possible DV?

1. **Taking it further**

Once DV has been raised as a topic, how much do you probe/ how far do you go?

Do you feel confident/ comfortable talking in greater depth about DV?

What do you do with information you have been given? (safeguarding, referring on, adding to crisis plan, impact on risk assessment/ decision-making)

Have you ever made an onwards referral in relation to DV? If so how many and where to?

Are you aware of third sector support services related to DV in your area?

1. **Knowledge and experience of DV**

Do you think it is important to ask? If yes, why is that? And if no, why not?

Do you feel knowledgeable/ experienced in talking about DV?

Have you completed any learning about DV? (e.g. from personal interest? A training course provided by your hospital/ professional body? or while you were training to be a clinician at University or College?)

Is there anything specific that would help you feel more confident/ comfortable?

1. **Training**

Asking about DV is recommended by NICE as an important part of mental health assessment following incidents of SH/SA. Do you agree?

How much is DV as a topic valued in your organisation? E.g., is it recorded in your notes?

Do you feel having training or guidance on the topic of DV would be helpful?

If so, what might this look like?

1. **Other**

What are some of the other issues we might not have covered here in talking about DV?

**Are there any other important research areas that we should cover in the future?**
